# Immunosuppressant exposure confounds gene expression analysis in systemic lupus erythematosus

**DOI:** 10.3389/fimmu.2022.964263

**Published:** 2022-08-17

**Authors:** Melissa Northcott, Linden J. Gearing, Julie Bonin, Rachel Koelmeyer, Alberta Hoi, Paul J. Hertzog, Eric F. Morand

**Affiliations:** ^1^ Centre for Inflammatory Diseases, Monash University, Clayton, VIC, Australia; ^2^ Centre for Innate Immunity and Infectious Diseases, Hudson Institute of Medical Research, Clayton, VIC, Australia; ^3^ Department of Molecular and Translational Science, Monash University, Clayton, VIC, Australia

**Keywords:** systemic lupus erythematosus, transcriptional profiling, gene modules, immunosuppressants, glucocorticoids

## Abstract

**Objectives:**

The analysis of gene module expression in SLE is emerging as a tool to identify active biological pathways, with the aim of developing targeted therapies for subsets of patients. Detailed information on the effect of immunosuppressants on gene module expression is lacking. We aimed to examine the impact of medication exposure on gene module expression.

**Methods:**

A set of commercially available disease-relevant gene modules were measured in 730 whole blood samples from a dedicated lupus clinic on whom prospectively collected, contemporaneous clinical data including medication exposure were available.

**Results:**

Compared to heathy controls, SLE patients showed over-expression of IFN and under-expression of B cell, T cell and pDC modules. Neutrophil module over-expression and under-expression of B and T cell modules were observed in patients with active lupus nephritis or highly active disease (SLEDAI-2K > 8), while Lupus Low Disease Activity State (LLDAS) had inverse associations. Disease activity in other organ domains was not associated with specific gene modules. In contrast, medications were associated with multiple effects. Glucocorticoid use was associated with under-expression of T cell, B cell and plasmablast modules, and over-expression of neutrophil modules. Mycophenolate and azathioprine exposure were associated with plasmablast module and B cell module under-expression respectively. Disease activity associations with neutrophil over-expression and lymphocyte module under-expression were attenuated by multivariable adjustment for medication exposure.

**Conclusion:**

Medications have significant effect on gene module expression in SLE patients. These findings emphasize the need to control for medications in studies of gene expression in SLE.

## Introduction

Systemic lupus erythematosus (SLE) is a clinically diverse systemic autoimmune condition with a pathogenesis that is incompletely described. It is typically treated with broad spectrum immunosuppression, with a heavy reliance on glucocorticoids, due to a lack of alternative, more targeted options (1). Over the past decade, whole blood transcriptional profiling has emerged as a technique to help uncover biological pathways which are active in SLE patients (2), with the ultimate aim of identifying potential therapeutic targets in SLE, enabling more targeted treatments.

Due to the sheer volume of data generated from transcriptional profiling, gene “modules” have been developed that comprise groups of coordinately expressed transcripts that represent specific biological processes (3). To date, the upregulation of type 1 interferon (IFN) gene modules has been the most striking and consistent finding from blood transcriptional profiling in SLE (4, 5). However, other relationships have also been identified, including the over-expression of neutrophil modules in patients with active lupus nephritis (6), and a relationship between disease activity and plasmablast gene modules, particularly in African American patients (7). Attempts have also been undertaken to biologically cluster patients based on gene module expression profiles with the hope of developing personalized targeted therapies for subsets of patients with SLE, and work in this field is ongoing (8).

Patients with SLE who are studied for gene expression are generally taken from established cohorts and are on established therapy at the time of sampling. Thus, virtually all gene expression data from human subjects with SLE is potentially influenced by medication exposure. Despite this, the effect of medications on gene module expression in SLE has not been comprehensively examined. A better understanding of the effect of medications in SLE on gene expression is required in order to better interpret these data as we move forward in our endeavor to understand this disease. Here, we analyses a selection of disease-relevant gene modules in a large cohort of SLE patients whose clinical features, including medication use, were well characterized. Strikingly, we found that medications, in particular glucocorticoids, had a marked impact on gene module expression. These data strongly suggest that medication exposure needs to be carefully controlled for in studies using blood transcriptional profiling in SLE.

## Materials and methods

Consenting adult patients meeting American College of Rheumatology criteria for SLE (9) were recruited from the Monash Lupus Clinic, a specialized lupus management clinic at a large tertiary hospital in Melbourne, Australia between July 2017 and July 2019. Whole blood RNA samples were collected in PAXgene^®^ tubes at the time of routine pathology collection. Extensive clinical data temporally relating to blood sampling were prospectively collected using standardized data collection formats (10), including demographic data, medication use, SLE disease activity index (SLEDAI-2K) (11), SELENA SLEDAI flare index (SFI) (12) and Lupus Low Disease Activity State (LLDAS) (13) status. Patients were classified as having a past organ manifestation if they were documented to have met the relevant organ criteria by either the ACR criteria for SLE (9) or the Systemic Lupus International Collaborating Clinics (SLICC) Classification Criteria for SLE (14) on enrolment to the registry. Control samples were collected from consenting adults with no known diagnosis of autoimmune disease. The project was approved by the Human Research Ethics Committee of Monash Health.

Expression of commercially available IFN, IFN beta, IFN gamma, plasmablast, neutrophil, plasmacytoid dendritic cell (pDC), B cell and T cell gene modules was measured by Dxerity, Rancho Dominguez, CA USA, using chemical ligation-dependent probe amplification and gene expression analysis by capillary electrophoresis as described (5).

Statistical analysis was performed using R software. The limma package (v3.50.0) was used to perform module differential expression analyses (15, 16). Initially, all SLE samples were compared to the healthy controls. Subsequent univariate and multivariate analyses examined module expression in the SLE samples in the context of different clinical variables. For each of these analyses, all SLE samples were included, unless they had any missing values for the variables under consideration.

For all analyses, a design matrix was creating incorporating the relevant variables. Since some samples were from the same donor, duplicate correlation was calculated using donor as a blocking factor. A linear model was fit using the lmFit function with the module expression values, design matrix, blocking factor and consensus correlation. Empirical Bayes moderated *t*-tests were performed and p-values obtained using the eBayes function. For each analysis, module p-values were adjusted separately for multiple testing. The top Table function was used to obtain 95% confidence intervals. For variables of interest, module log_2_ fold changes were plotted as points with 95% confidence intervals as horizontal bars. Significant modules were colored according to the direction of change (red if up, blue if down). The sinaplot package (v1.1.0) was used to plot module expression values for individual samples, grouped by disease status and further sub-divided by other clinical parameters, if relevant (17). For plots associated with multivariate analyses including medication exposure, samples were colored by dose (missing values were indicated by open circles). The pheatmap package (v1.0.12) (18) was used to generate a heat map of log_2_ fold changes associated with clinical variables from the univariate analyses, compared to the relevant reference value (for example, males versus females, low complement versus normal complement). Any module with a significant adjusted p-value was indicated as follows: * p < 0.05, ** p < 0.01, *** p < 0.001.

## Results

801 samples from 210 SLE patients were collected across the study period. 730 samples from 205 patients passed quality control and were used in the analysis. 91.2% were female with a median [range] age of 43[18-84] at study entry. 41.0% of participants were of European and 39.5% of Eastern Asian background. Further demographic details are provided in **Table 1**. Disease manifestations varied across the population (**Table 1**), with the most common manifestations being arthritis (67.8%), skin disease (56.1%), leukopenia (50.2%) and renal disease (43.9%). Medication data were available for 701 samples from 200 patients. 293 (41.8%) of samples were from patients taking low dose glucocorticoids (1.0-7.5mg prednisolone/day) and 111 (15.9%) were from patients taking >7.5mg prednisolone/day. 294 (41.9%) samples were from patients taking mycophenolate and 110 (15.7%) samples were from patients taking azathioprine. 625 (89.1%) samples were from patients taking hydroxychloroquine. Further details of medication exposure are shown in **Table 1**. Samples from 41 healthy controls were also collected for comparison. Demographic data for these people are also shown in **Table 1**.

**Table 1:** Patient and control demographics and patient disease manifestations.

	SLE	*Control*
Number of subjects	205	*41*
Sex (M:F)	18:187	*11:30*
European ethnicity (n (%))	84 (41.0%)	*26 (63.4%)*
SE/NE Asian ethnicity (n (%))	81 (39.5%)	*11 (26.3%)*
Southern/Central Asian ethnicity (n (%))	16 (7.8%)	*2 (4.9%)*
Other/unknown ethnicity (n (%))	24 (11.7%)	*2 (4.9%)*
Age at study entry (years) (median [range])	43[18-84]	*38[24-61]*
Age of disease onset (years) (median [range])	28[7-77]	
Duration of disease (years) (median [range])	10 [0-49]	
Number of samples/patient (median [range])	3[1-17]	
**Disease manifestations (past)**		
Renal disease (n (%))	90 (43.9%)	
Arthritis (n (%))	139 (67.8%)	
Skin disease (n (%))	115 (56.1%)	
Leukopenia (n (%))	103 (50.2%)	
Thrombocytopenia (n (%))	16 (7.8%)	
Haemolytic anaemia (n (%))	20 (9.8%)	
Serositis (n (%))	71 (34.6%)	
Neurological disease (n (%))	25 (12.2%)	
ANA positive (n (%))	200 (97.6%)	
Anti-dsDNA (n (%))	Positive 160 (78.0%) Negative 35 (17.1%) ND* 10 (4.9%)	
Anti-RNP (n (%))	Positive 55 (26.8%) Negative 132 (64.4%) ND 18 (8.7%)	
Anti-Ro (n (%))	Positive 101 (49.2%) Negative 85 (41.5%) ND 19 (9.2%)	
Anti-La (n (%))	Positive 42 (20.5%) Negative 143 (69.8%) ND 20 (9.8%)	
Anti-Sm (n (%))	Positive 34 (16.6%) Negative 151 (73.7%) ND 20 (9.8%)	
Low complement (n (%))	Positive 174 (84.9%) Negative 18 (8.8%) ND 13 (6.3%)	
**Disease activity at time of sample collection**	**Number of patients**	**Number of samples**
Any active renal disease (n (%))	49 (23.9%)	197 (27.0%)
Proteinuria (n (%))	45 (30.0%)	188 (25.8%)
Haematuria (n (%))	18 (8.8%)	75 (10.3%)
Arthritis (n (%))	18 (8.8%)	33 (4.5%)
Serositis (n (%))	4 (2.0%)	4 (0.5%)
Skin disease (n (%))	35 (17.1%)	57 (7.8%)
Any haematological (n (%))	32 (15.6%)	72 (9.9%)
Leukopenia (n (%))	24 (11.7%)	58 (7.9%)
Haemolysis (n (%))	1 (0.4%)	1 (0.1%)
Thrombocytopenia (n (%))	9 (4.4%)	14 (1.9%)
Neurological disease (n (%))	1 (0.4%)	4 (0.5%)
Low C3/C4 (n (%))	145 (70.7%)	478 (65.5%)
High anti-dsDNA (n (%))	127 (62.0%)	452 (61.9%)
**Medication use at time of sample collection** *(data available for 701 samples from 200 patients)*	**Number of patients**	**Number of samples**
Hydroxychloroquine (n (%))	178 (89.0%)	625 (89.1%)
Azathioprine (n (%))	43 (21.5%)	110 (15.7%)
Mycophenolate (n (%))	67 (33.5%)	294 (41.9%)
Methotrexate (n (%))	25 (12.5%)	93 (13.3%)
Cyclophosphamide (n (%))	6 (3.0%)	13 (1.9%)
Belimumab (n (%))	3 (1.5%)	15 (2.1%)
Rituximab (n (%))	12 (6.0%)	19 (2.7%)
Prednisolone 0 mg (n (%))	114 (57.0%)	298 (42.5%)
Prednisolone 1-7.5 mg (n (%))	89 (44.5%)	293 (41.8%)
Prednisolone >7.5 mg (n (%))	37 (18.5%)	111 (15.9%)

*ND, not documented.

**Figure 1:**
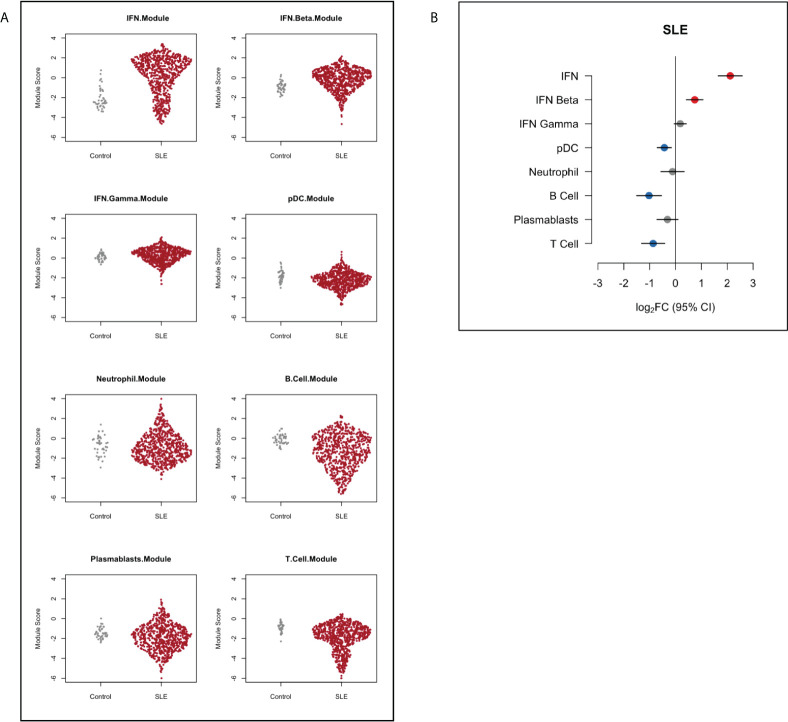
Gene module expression in SLE patients compared to healthy controls depicted **(A)** as dot plots and **(B)** as relative log_2_ fold change (log_2_FC). Blue symbols represent modules that were significantly under-expressed, grey symbols modules that were not significantly different and red symbols represent modules that were significantly over-expressed in SLE patients compared with controls. Horizontal bars indicate 95% confidence intervals (95%CI).

First, we compared gene module expression in SLE patients to healthy controls. SLE patients showed over-expression of IFN modules (log_2_FC 2.11, 95%CI (1.65 to 2.58), p=<0.001). B cell, T cell and pDC modules were under-expressed in SLE patients compared to healthy controls (B cell log_2_FC -1.02, 95%CI (-1.51 to -0.54), p=0.002; T cell log_2_FC -0.87, 95%CI (-1.32 to -0.42), p=0.0003; pDC module log_2_FC -0.43, 95%CI (-0.71 to -0.16), p=0.002) (**Figure 1**).

**Figure 2:**
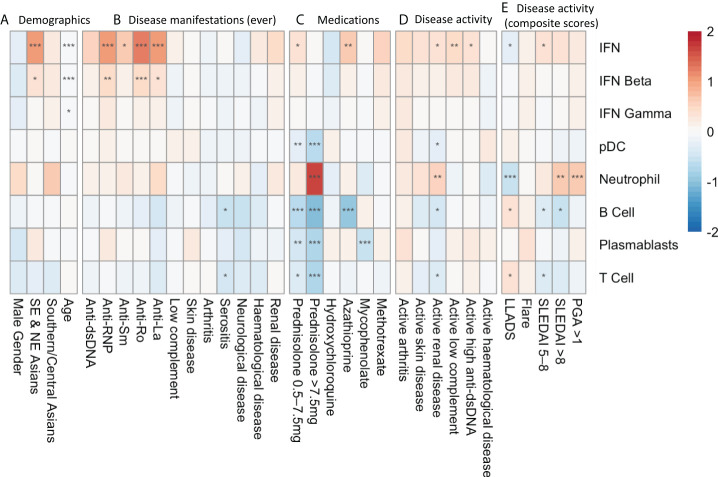
Univariate analysis depicting log_2_FC gene expression in SLE patients associated with **(A)** demographic factors, **(B)** disease manifestations at any time point in their documented disease course, **(C)** medication exposure at the time of sample collection, **(D)** SLEDAI-2K organ domain disease activity at time of sample collection and **(E)** composite disease activity measurements at the time of sample collection. * p<0.05 **p<0.01 ***p<0.001. Asian ancestry backgrounds are compared to European ancestry.

Before assessing medication effects on gene expression modules, we performed univariate analysis to identify associations between gene modules and demographic and clinical variables (**Figure 2**). We have previously reported associations between clinical variables and type 1 IFN signature expression in this cohort (5) however these results are included in **Figure 2** for visual comparison to other modules. The IFN beta module followed a similar pattern of association with clinical variables as the IFN module, with over-expression in patients of Eastern Asian heritage (log_2_FC 0.31, 95%CI (0.07-0.55), p=0.012), under-expression in patients of older age (log_2_FC -0.02/per year, 95%CI (-0.02 to -0.01), p=<0.001), and an association with anti-RNP (log_2_FC 0.44, 95%CI (0.19-0.69), p<0.001), anti-Ro (log_2_FC 0.44, 95%CI (0.20-0.67), p< 0.001), and anti-La (log_2_FC 0.39, 95%CI (0.11-0.67), p=0.006) autoantibodies. There was no association between the IFN gamma module and any disease manifestation, however lower IFN gamma expression was also associated with increased age (log_2_FC -0.01/per year, 95%CI (-0.01 to 0.00), p=0.032).

There were no associations with historical organ manifestation and gene module expression, with the exception of ever having serositis, which was associated with lower expression of B cell (log_2_FC -0.57, 95%CI (-0.93 to -0.22), p=0.011) and T cell (log_2_FC -0.44, 95%CI (-0.77 to -0.10), p=0.041) modules.

Active renal disease (any score in the renal SLEDAI-2K domains) was associated with increased expression of the neutrophil module (log_2_FC 0.51, 95%CI (0.23 to 0.79), p=0.0027), and suppression of the B cell (log_2_FC -0.45, 95%CI (-0.74 to -0.15), p=0.015), T cell (log_2_FC -0.37, 95%CI (-0.65 to -0.09), p=0.021) and pDC modules (log_2_FC -0.21, 95%CI (-0.38 to -0.04), p=0.028). Disease activity in other organ domains assessed by SLEDAI-2K domain scores did not show associations with specific gene modules.

We did not find an association between gene module expression and disease flares measured using the SFI. In contrast, highly active disease defined as SLEDAI-2K>8 was associated with neutrophil module overexpression (log_2_FC 0.66, 95%CI (0.31 to 1.01), p=0.002), as was PGA >1.0 (log_2_FC 0.63, 95%CI (0.33 to 0.92), p=<0.001). Correspondingly, neutrophil module overexpression was negatively associated with being in LLDAS (log_2_FC -0.57, 95%CI (-0.35 to -0.79), p<0.001). Increased SLEDAI-2K and LLDAS were also associated positively and negatively, respectively, with suppressed B and T cell module expression (**Figure 2**).

Medications had a potent impact on gene module expression in univariate analysis (**Figure 2**). Prednisolone use >7.5mg/day at the time of sample was associated with increased expression of the neutrophil module (log_2_FC 1.63, 95%CI (1.31- 1.96, p<0.001). Any prednisolone use was also associated with suppression of the T cell, B cell, plasmablast and pDC modules.

Mycophenolate use was associated with suppression of the plasmablast module (log_2_FC -0.55, 95%CI (-0.80 to -0.29, p<0.001), and azathioprine was associated with B cell module suppression (log_2_FC -0.96, 95%CI (-1.36 to -0.57, p<0.001). Methotrexate and hydroxychloroquine use did not significantly alter gene module expression. The effects of prednisolone, mycophenolate, azathioprine and hydroxychloroquine on gene module expression at the patient level are depicted in **Supplementary Figure 1**.

All samples from all patients were included in the univariate analysis presented in **Figure 2**. To ensure there was not undue bias from this approach, we also performed this analysis using a single sample from each subject and noted similar patterns of expression (data not shown).

**Figure 3:**
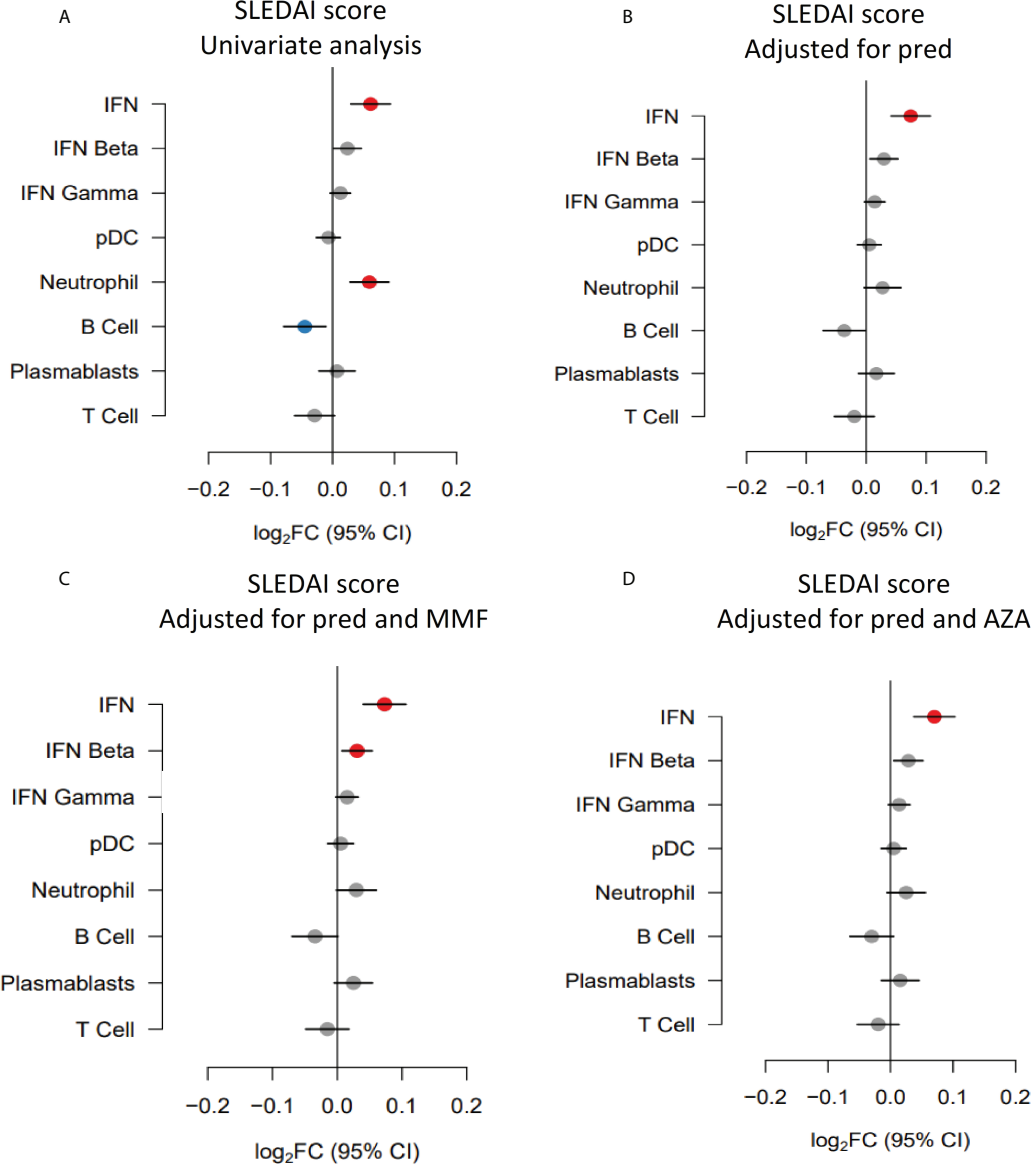
Gene module expression associations with disease activity (SLEDAI-2K) (expressed as log_2_FC per SLEDAI point) in **(A)** univariate analysis, **(B)** analysis adjusted for prednisolone dose (pred), **(C)** analysis adjusted for prednisolone and mycophenolate (MMF) and **(D)** analysis adjusted for prednisolone and azathioprine (AZA) exposure. Blue symbols represent statistically significant under-expression of gene module, grey symbols represent results that were not significant, and red symbols represent statistically significant over-expression.

As discussed above, overall disease activity was associated with increased neutrophil module expression and suppression of the B cell module in univariate analysis. Given that prednisolone also impacted on expression of these modules, we performed multivariate analysis. When adjusting for prednisolone dose, the relationships between both over-expression of the neutrophil module and under-expression of the B cell module in univariate analysis (**Figure 3A**) were attenuated (**Figure 3B**).

Previous studies have suggested that plasmablast module expression reflects disease activity in some patients. As mycophenolate was associated with suppression of the plasmablast module in univariate analysis, we performed a multivariate analysis of the association with disease activity adjusting for mycophenolate use. The relationship between the plasmablast module trended towards positivity after adjustment (log_2_FC 0.02, 95%CI (0.00 to 0.05), p=0.13) but did not reach statistical significance (**Figure 3C**). Similarly, adjusting for azathioprine exposure did not unveil any gene module expression relationships with disease activity (**Figure 3D**).

We next focused specifically on renal disease. Renal disease activity assessed using the renal SLEDAI-2K domains was associated with increased expression of the neutrophil module, and suppression of the pDC, B cell and T cell modules, as discussed above. Over-expression of the neutrophil module was also strongly associated with prednisolone usage, and suppression of the B cell module was associated with azathioprine use. We therefore performed multivariate analyses to investigate these relationships further.

**Figure 4:**
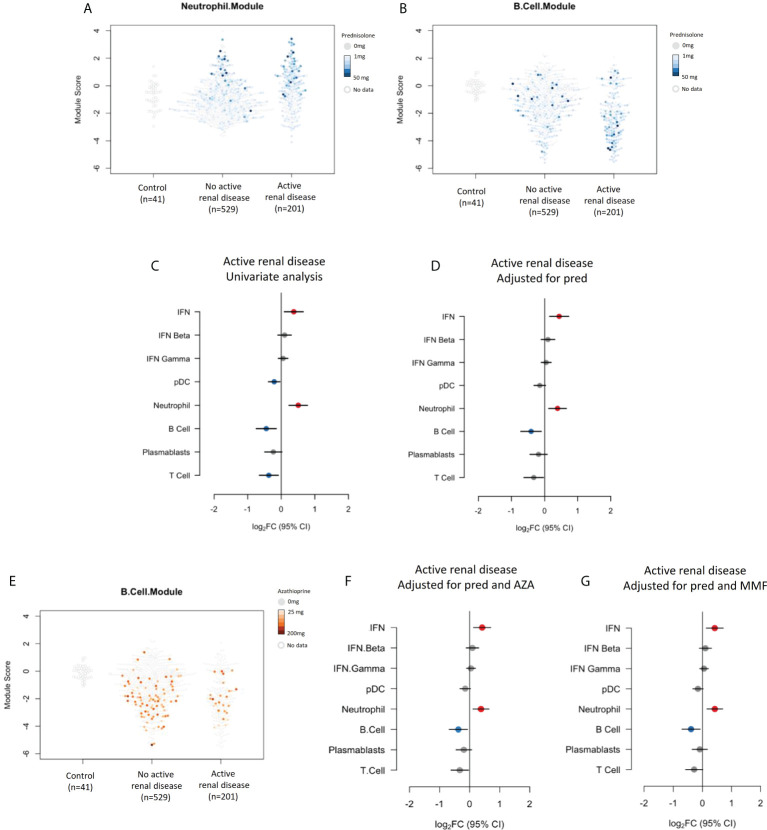
**(A)** Neutrophil module expression in patients with and without active renal disease and their associated prednisolone dose. **(B)** B cell module expression in patients with and without active renal disease and their associated prednisolone dose. **(C)** Expression of gene modules in patients with active renal disease compared to those without active renal disease in univariate analysis and **(D)** analysis adjusted for prednisolone exposure. **(E)** B cell module expression in patients with and without renal disease and their associated azathioprine use. **(F)** Expression of gene modules in patients with active renal disease compared to those without active renal disease adjusted for prednisolone and azathioprine (AZA) exposure and **(G)** prednisolone and mycophenolate (MMF) exposure.

Neutrophil and B cell module expression in individual patients with and without active renal disease and their associated prednisolone dose are shown in **Figure 4A, B** respectively. The relationship between neutrophil module overexpression with active renal disease seen in univariate analysis (**Figure 4C**) remained significant when correcting for prednisolone dose (log_2_FC 0.39, 95% CI (0.12 to 0.66), p=0.018), as did the association with B cell module suppression (log_2_FC -0.41, 95% CI (-0.72 to -0.10), p=0.024), (**Figure 4D**). B cell module expression in individual patients with and without active renal disease and their associated azathioprine dose are shown in **Figure 4E**. Including azathioprine in the multivariate analysis did not alter the relationships between renal disease activity and gene module expression compared to controlling for prednisolone alone (**Figure 4F**). We also performed an analysis adjusting for mycophenolate use given that patients with renal disease activity commonly receive this medication. Adjusting for mycophenolate use did not alter the associations of active renal disease with neutrophil and B cell module expression (**Figure 4G**).

**Figure 5:**
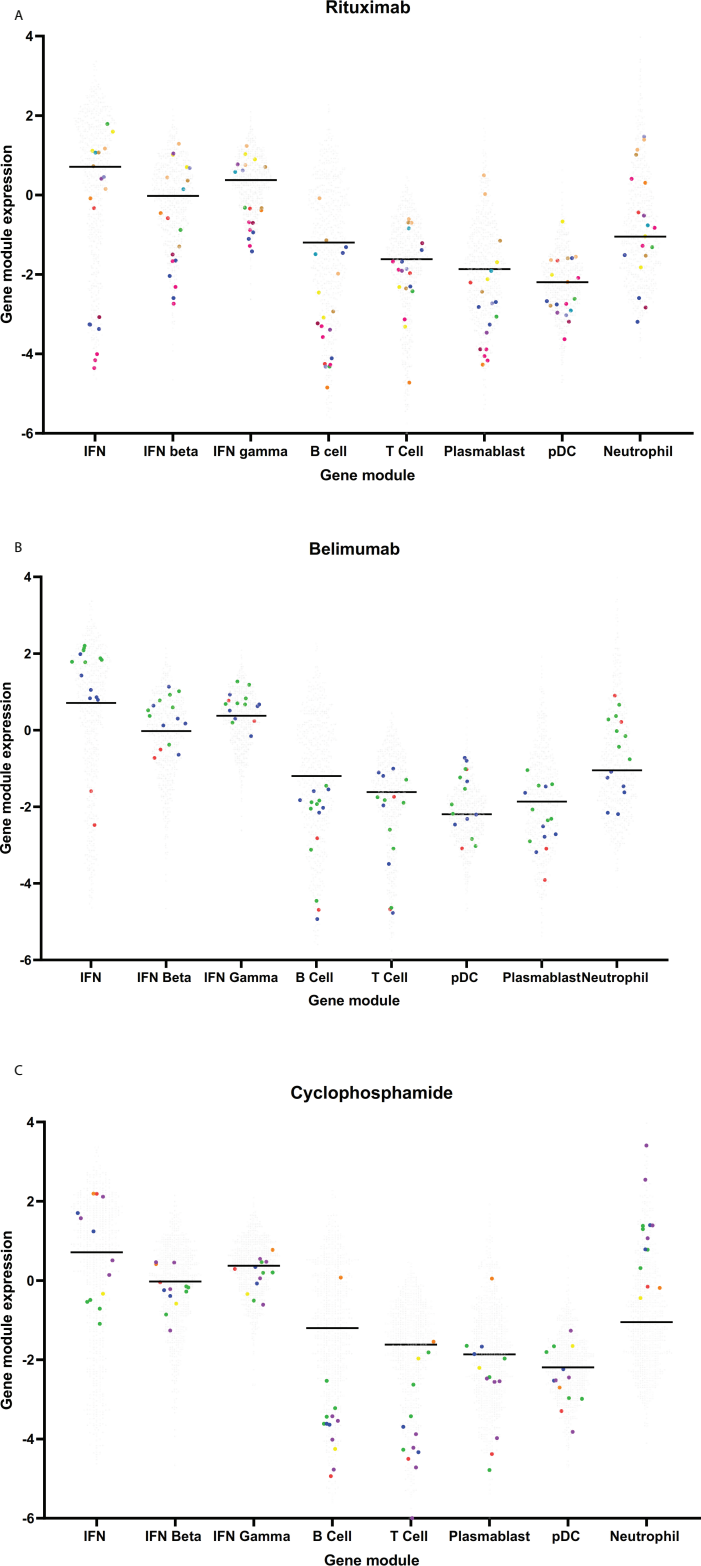
Gene module expression in patients taking **(A)** rituximab, **(B)** belimumab and **(C)** cyclophosphamide. Grey dots represent samples from patients not taking these medications, colored dots represent samples taken from patients taking these medications. On each graph, each color represents samples from a single patient.

Small numbers of samples from patients exposed to rituximab (19 samples from 12 patients), belimumab (15 samples from 3 patients) or cyclophosphamide (13 samples from 6 patients) at the time of sample collection were analyzed. The number of samples from patients exposed to these medications were too few to be included in a multivariate analysis, however univariate analyses were performed and gene module expression in these samples compared to the cohort as a whole is depicted visually in **Figure 5**. Not unexpectedly, samples from patients who had received rituximab in the six months prior to their blood sample had suppressed expression of the B cell module compared to patients not exposed to rituximab (log_2_FC -1.30, 95%CI (-2.10 to -0.55), p=0.0057). Belimumab did not have any significant associations with gene module expression in this cohort (**Figure 5B**), however as patient numbers are small this needs to be interpreted with caution. Samples from patients who received cyclophosphamide in the last 6 months also had under-expression of the B cell module (log_2_FC -1.12, 95%CI (-1.82 to -0.41), p=0.0051), as well as the T cell module (log_2_FC -1.17, 95%CI (-1.84 to -0.50), p=0.0024), and over-expression of the neutrophil module (log_2_FC 1.6, 95%CI (0.98 to 2.27), p<0.001) (**Figure 5C**).

## Discussion

Gene module expression analysis is an emerging technique to help describe biological patterns in illnesses such as SLE. In this study, we examined the effects of medications commonly prescribed in SLE on gene module expression and explored relationships between medications and clinical disease activity with gene modules. We found strong effects on gene module expression in patients taking glucocorticoids, as well as suppression of the plasmablast module in patients taking mycophenolate and B cell module in patients taking azathioprine. Adjusting for exposure to medications, particularly prednisolone, attenuated some relationships between disease activity and gene modules suggested in univariate analysis. These results highlight the importance of recording detailed medication exposure and adjusting for this when analyzing gene expression patterns in SLE patients who have heterogenous medication exposure.

Several studies examining gene module expression in SLE patients have been published in recent years. In keeping with our results, over-expression of IFN and under-expression of lymphocyte modules compared to healthy control patients has been consistently reported (3, 6, 7). Some studies have previously touched on medication effects on gene expression in SLE. Guthridge et al. published a study using DNA microarray expression in 198 adult SLE patients (8). Of note, patients who had taken rituximab, cyclophosphamide or IV steroids within one year of the study were not included. This study reported seven clusters of patients who expressed similar gene module patterns, but reported no clustering in association with overall disease activity, or organ involvement. However, interestingly they did report significant medication differences across the modules, particularly in relation to glucocorticoids and mycophenolate. In keeping with our work in adult SLE patients, a large study in pediatric SLE patients by Banchereau et al. reported overexpression of the neutrophil module in patients taking glucocorticoids, and also suppression of the plasmablast signature in patients taking either mycophenolate and cyclophosphamide (7). A paper by Jourde-Chiche *et al.* also showed glucocorticoid usage was associated with overexpression of the neutrophil module (6). Our research expands on this work by focusing on these relationships in more detail, and illustrating the magnitude of effects immunosuppressive medications can have on gene expression.

Interestingly, although several studies have reported associations between the plasmablast module and disease activity in SLE populations, we did not replicate this finding in our study (7, 8). Both glucocorticoids and mycophenolate suppressed the plasmablast signature in our population, but correcting for these medications in our analysis did not unveil a significant relationship with disease activity. This correlation was shown to be strongest in African-American patients in one study (7), a population that was absent from our cohort which was predominantly patients of European and Asian ancestry. Previous gene module studies in SLE patients of Asian ancestry are limited.

The association between renal disease activity in SLE and over-expression of neutrophil gene expression modules has been reported both in adult and pediatric populations (6, 7, 19). Given the well-known effect of glucocorticoid use on peripheral blood neutrophil counts, and the strong association between the neutrophil module and glucocorticoid exposure we observed, we explored this relationship further. Overexpression of the neutrophil module in patients with active renal disease remained significant upon adjusting for glucocorticoid exposure. B cell module under-expression also remained significant, however other module expression associations with renal disease that were noted in the univariate analysis were lost when adjusted for steroid exposure. We were interested also to note overexpression of the neutrophil module in the small subset of patients receiving cyclophosphamide therapy. This cohort was too small to be included in a multivariate analysis, however we hypothesize that this could be related to active renal disease and concurrent glucocorticoid exposure in this group.

There are several limitations to this work. Whilst this is a large cohort of patients, subsets of patients taking some medications were small, which limited the ability to analyze these medications in detail. Although clinical data were collected prospectively, our data were analyzed cross-sectionally, and the relationships described are therefore associative as opposed to causative. Further studies analyzing samples from patients before and at specific times points after taking medications of interest may provide further information. We report the results of a finite number of disease-relevant gene modules described in the literature and available in a commercial assay system. However, we believe this work provides proof of concept of the significant impact of medications on gene module expression in SLE, and therefore has important implications for future studies. Collaboration with other groups who have performed transcriptional profiling on SLE patient cohorts could be considered to examine the effects of medications on gene modules beyond those analyzed in this study.

In summary, the study of gene module expression in SLE patients has become an important tool in the quest to uncover pathophysiological processes occurring in this disease, and potentially to target specific therapies to specific patients. Our findings indicate that medications commonly used in the treatment of SLE, particularly glucocorticoids, significantly impact on gene module expression, and adjusting for medication use attenuated apparent clinical associations seen in unadjusted analysis. This highlights the importance of carefully controlling for medication use in research utilizing gene expression analysis techniques in humans with SLE.

## Data availability statement

The raw data supporting the conclusions of this article will be made available by the authors, without undue reservation.

## Ethics statement

The studies involving human participants were reviewed and approved by Monash Health Human Research Ethics Committee, Monash Health, Clayton, Victoria, Australia. The patients/participants provided their written informed consent to participate in this study.

## Author contributions

MN performed the literature review. MN, LG, PH and EM were involved in study design. MN and LG analyzed the data. AH was involved in patient recruitment and management of the Australian Lupus Registry and Biobank. JB and RK were involved in sample management and extraction of data from the Australian Lupus Registry. MN and EM wrote the manuscript. MN and LG had access to and verified all data. MN and EM had the final responsibility for the decision to submit for publication. All authors contributed to the article and approved the submitted version.

## Funding

This study was supported by grants from the Lupus Research Alliance, New York [Grant 381308 to EM] and the National Health and Medical Research Council (NHMRC), Australia [Scholarship 1133155 to MN]. The gene module expression assays were funded by DxTerity Diagnostics, Rancho Dominguez, CA USA. The Australian Lupus Registry and Biobank has received sponsorship from Arthritis Victoria, AstraZeneca, UCB and Janssen. The funders were not involved in the study design, collection, analysis, interpretation of data, the writing of this article or the decision to submit it for publication.

## Conflict of interest

Outside the scope of this work, EM has received consulting fees from AbbVie, AstraZeneca, Amgen, Biogen, Bristol Myers Squibb, Eli Lilly, EMD Serono, Genetech, Janssen, Servier and UCB, and research grants from AbbVie, AstraZeneca, Bristol Myers Squibb, Eli Lilly, EMD Serono and Janssen. AH has received research grants from AstraZeneca, Merck and GSK, and consulting fees from Abbvie, AstraZeneca, BMS, GSK, Janssen and Pfizer.

The remaining authors declare that the research was conducted in the absence of any commercial or financial relationships that could be construed as a potential conflict of interest.

## Publisher’s note

All claims expressed in this article are solely those of the authors and do not necessarily represent those of their affiliated organizations, or those of the publisher, the editors and the reviewers. Any product that may be evaluated in this article, or claim that may be made by its manufacturer, is not guaranteed or endorsed by the publisher.
