# Supplementary material for: Immunosuppressant exposure confounds gene expression analysis in systemic lupus erythematosus
Source: Front Immunol. 2022 Aug 17;13:964263. doi: 10.3389/fimmu.2022.964263 (PMC9430375; doi:10.3389/fimmu.2022.964263)
Supplement: Supplementary file 1 [file Image_1.pdf]

Supplementary Data

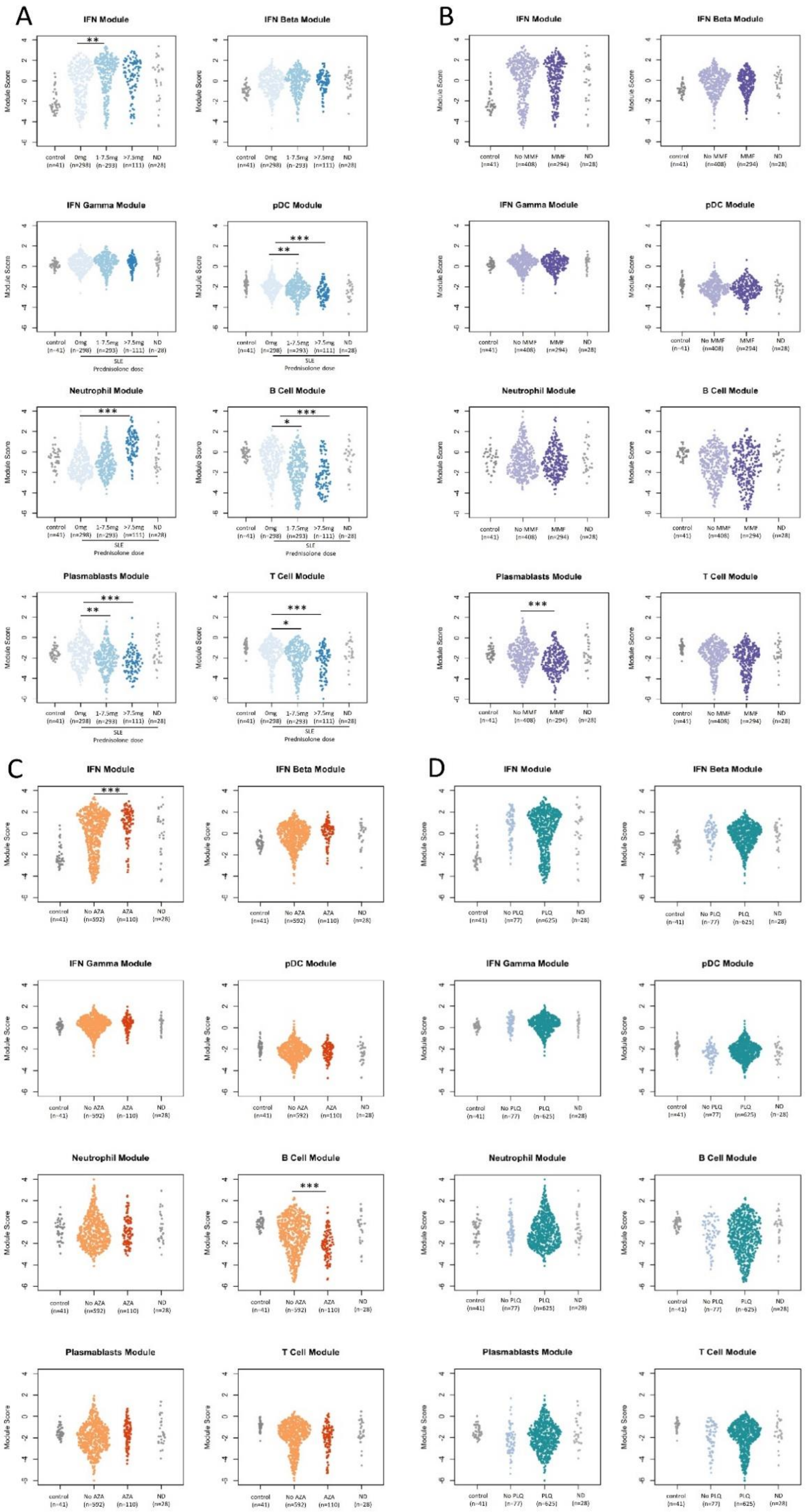

Figure S1 Gene expression in healthy controls and patients taking and not taking A) prednisolone, B) mycophenolate, C) azathioprine and D) hydroxychloroquine. ND=no data. * p<0.05 **p<0.01 ***p<0.001
